# Navigating the Labyrinth: Organizational Challenges in Integrated Diabetes Care for Individuals with Type 2-Diabetes and Schizophrenia: A Qualitative Exploration from the Healthcare Professionals’ Perspective

**DOI:** 10.5334/ijic.8621

**Published:** 2025-10-31

**Authors:** Tanja Juhl Mikkelsen, Dorte Moeller Jensen, Elsebeth Stenager, Mette Juel Rothmann

**Affiliations:** 1 Steno Diabetes Center Odense, Odense University Hospital, Odense, Denmark; 2 Department of Clinical Research, University of Southern Denmark, Odense, Denmark; 3 Psychiatric Research Unit, Aabenraa, Department of Regional Health Services Research, University of Southern Denmark, Denmark; 4 Department of Regional Health Research, University of Southern Denmark, Odense, Denmark

**Keywords:** co-occurring schizophrenia, type 2 diabetes, dual diagnosis, integrated care, holistic perspective

## Abstract

**Introduction:**

This study explores the challenges faced by healthcare professionals in providing integrated care for individuals living with co-occurring schizophrenia and type 2 diabetes in Denmark. Despite the increased complexity of managing both conditions, little research has focused on healthcare professionals experiences and the organizational challenges involved in delivering integrated care.

**Description:**

Using a qualitative exploratory design, the study involved semi-structured interviews with four healthcare professionals, 17 field observations of outpatient consultations, and one focus group interview, conducted between August 2020 and February 2021. Ricoeur’s interpretive philosophy was used for data analysis, focusing on the healthcare practices and challenges identified by healthcare professionals.

**Discussion:**

Three key themes emerged: (1) Specialization leads to lack of knowledge and fragmented care, (2) The need for guiding in a complex healthcare system, and (3) The missing link – a lack of overview of healthcare services. These challenges impact integrated care delivery, as discussed in the context of Denmark’s healthcare system and international evidence.

**Conclusion:**

The study highlights the need for a more integrated, holistic approach to care, including improved collaboration between specialized and general healthcare professionals, better care coordination, and policy improvements. Further research is needed to identify effective strategies for overcoming these barriers and to create more integrated care pathways for patients with complex conditions.

## Introduction

Schizophrenia is a severe mental illness (SMI) affecting approximately 27,700 individuals in Denmark. Although it impacts a relatively small proportion of the population, people with schizophrenia face substantially higher rates of acute somatic and psychiatric hospitalizations [1]. When combined with type 2 diabetes (T2D), the health burden increases markedly, as the prevalence of T2D among individuals with schizophrenia is estimated at 10–15% [2,3]. This comorbidity contributes to excess mortality, shortening life expectancy by 15–20 years compared to the general population [1,4], and imposes a considerable strain on healthcare systems and society at large [5,6].

Caring for individuals with both schizophrenia and T2D presents significant complexities, amplified by systemic obstacles within the healthcare system. Healthcare professionals (HCPs) often struggle to coordinate services due to sectoral silos, insufficiently person-centered approaches, and the challenge of managing physical health alongside ongoing psychiatric care [7,8]. Patients with both conditions typically require frequent interactions with multiple HCPs, yet the lack of integration across sectors often results in disjointed and uncoordinated care [8,9]. In addition, limited collaboration between specialists, general practitioners (GPs), and mental health professionals, as well as stigma toward people with schizophrenia, further compounds these difficulties [10,11].

In Denmark, treatment responsibilities are distributed across several sectors. Psychiatric care is generally delivered in regional outpatient clinics, while T2D is primarily managed in general practice and through municipal health services. Municipalities also provide rehabilitation and social support. In more complex cases, diabetes treatment is referred to specialized hospital-based diabetes centers. This division reflects a broader structural separation between somatic and mental healthcare, often resulting in poor communication and minimal coordination between providers. At present, no formal integrated care pathway exists for people managing both conditions. Instead, care is frequently parallel and fragmented, with limited information exchange, a lack of shared treatment plans or care coordinators, and a significant burden placed on patients and their families to bridge these gaps.

Such organizational barriers make it particularly difficult for HCPs to deliver cohesive, coordinated care. The absence of a clear, unified care structure, combined with the demands of managing two complex chronic conditions, limits the ability of professionals to provide comprehensive support [7,12,13].

Consequently, affected individuals often encounter numerous healthcare contacts across disciplines, including GPs, psychologists, and psychiatrists, without sufficient continuity or collaboration between services [1].

Although previous studies have documented the difficulties of treating patients with concurrent mental and physical illnesses, such as poor integration and limited service access [10,14,15], only a few have specifically explored how HCPs experience and manage care for individuals with both schizophrenia and T2D [7,16,17].

Integrated care models are increasingly acknowledged as essential for supporting patients with complex co-occurring conditions like these [18]. However, despite growing recognition of this need, healthcare systems still face considerable difficulties in bridging the divide between sectors due to increasing specialization and weak cross-sector collaboration. This study investigates how systemic and organizational challenges influence the provision of integrated care for individuals with schizophrenia and T2D. By examining the experiences of HCPs navigating this complexity, we aim to identify opportunities for improving care coordination. In doing so, the study contributes to broader discussions on advancing health outcomes through better communication and collaborative practice at the intersection of mental and physical health.

## Ethical approval

Ethical approval was obtained from the Danish Data Protection Agency (journal number: 19/45675) and was reported to the Committee on Health Research Ethics in the Region of Southern Denmark.

## Description of the approach used

This study adhered to the Standards for Reporting Qualitative Research (SRQR) [19], ensuring that the study was conducted with the highest standards of qualitative research.

## Methods

The study was part of a broader research project which, through a Participatory Design approach, aimed to explore the existing challenges of integrated care for individuals with both conditions and healthcare perspectives, as well as to co-design and co-create a new intervention informed by the users. Results from a patient perspective have been published elsewhere [10], describing how patients provided their informed consent to participate in the field observations referenced in this study.

This sub-study employed a qualitative exploratory research design to investigate the experiences of HCPs managing treatment and care for individuals with schizophrenia and T2D. This design was chosen to provide an in-depth understanding of current care practices in the field.

A dual-method approach, including seventeen field observations of outpatient consultations [20], four semi-structured individual interviews [21], and one focus group interview [22], was conducted. These methods served to uncover the lived experiences of HCPs, enabling a deep exploration of the challenges they face in delivering care to individuals with comorbid conditions.

The study adopted a phenomenological-hermeneutic approach, drawing inspiration from Paul Ricoeur’s philosophy, which allowed for both the description and interpretation of lived experiences. The phenomenological aspect of the study focused on capturing the detailed lived experiences of HCPs, while the hermeneutic approach enabled a deeper understanding through interpretation of these experiences [23,24].

### Setting and participants

HCPs involved in the care of individuals with both conditions were purposively sampled from two large outpatient clinics in psychiatry and diabetes in the Region of Southern Denmark between August 2020 and February 2021. Purposive sampling aimed to capture a representative view of the different HCPs involved in the treatment of individuals with both schizophrenia and T2D. This included a broad range of professional groups, such as both psychiatric and somatic experts, including doctors (N = 1 medical doctor from psychiatry), nurses (N = 1 diabetes nurse, 4 psychiatric nurses), and one occupational therapist. Additionally, general practitioners were included (N = 2), as they often bear significant responsibility for the coordination and follow-up of patients with complex comorbid health conditions. The goal was to ensure a comprehensive and nuanced understanding of treatment practices across different specialties and levels of clinical experience. One of the psychiatric nurses participated in both an individual interview and the focus group interview.

Due to the restrictions imposed by the COVID-19 pandemic, modifications to the study design were necessary. Some field observations were conducted outdoors where appropriate, and interviews were arranged online or by telephone to ensure participant safety and allow data collection to continue without interruption.

### Data Collection

The first author, a registered nurse and Ph.D. candidate specializing in diabetes, conducted the field observations and interviews. Despite limited direct experience in psychiatry, the first author had previously engaged in research at the intersection of psychiatry and diabetes, with publication in this area [7,10,25]. Furthermore, the study had the full support of a supervisory team and co-authors, including a professor specializing in psychiatry, to ensure extensive expertise within the psychiatric field.

The data collection consisted of field observations, semi-structured interviews, and one focus group interview, all aimed at exploring clinical practices and challenges in managing care for individuals with both schizophrenia and T2D.

Observations took place during regular appointments in either psychiatric or somatic settings. The first author was notified by HCPs prior to scheduled appointments with individuals having both conditions. Some patients were informed beforehand about the author’s presence.

Informed consent was obtained from all patients and HCPs prior to participation [26].

Maintaining an open mindset, the first author engaged in interactions, communication, and discussions to capture the participants’ lived experiences [20]. To ensure systematic observation, an observational guide based on Spradley’s nine dimensions [27] was employed. Field notes were not written during consultations to avoid distraction and discomfort. Instead, notes and quotations were transcribed immediately following the consultations. Active listening, mental note-taking, and reflective practices ensured comprehensive data collection [20].

Insights from these observations informed the development of the semi-structured interview guides used in the subsequent interviews.

Two semi-structured interview guides (Appendix 1), covering challenges in care for individuals with schizophrenia and T2D, were developed and pilot-tested with a nurse and a doctor before use in four individual interviews (two online, two by telephone, each lasting 30–60 minutes) and one face-to-face focus group interview (lasting 90 minutes). HCPs were e.g. asked: *“What do you find most challenging for the patient with schizophrenia in having to manage diabetes at the same time? If we assume that anything could be possible, is there anything you would like to do? Or do differently compared to usual practice? If so, please elaborate”*.

Participants were encouraged to share their experiences and suggestions from a narrative perspective, with follow-up questions focusing on what they found most important. All interviews were audio recorded and transcribed verbatim by the first author. Drawing insights from the field observations, a flexible interview approach was adopted to align with participants’ narratives. Sampling was discontinued once a certain level of repetition in HCPs’ experiences was identified.

The transcriptions and field notes formed the basis for a subsequent in-depth phenomenological-hermeneutic analysis aimed at interpreting and understanding the lived experiences of HCPs.

### Data analysis

The verbatim transcriptions and field notes were imported into NVivo version 12 for data analysis. The first author, in close collaboration with the supervisory team, conducted the analysis inspired by Paul Ricoeur’s philosophy of narrative and critical interpretation. This phenomenological-hermeneutic approach aimed to generate new understanding by interpreting lived experiences through a structured and reflective lens [23,24].

Ricoeur’s framework guided the interpretation process through three levels:

### Naive Reading

This initial phase involved reading all transcripts and field notes as one coherent text to gain an immediate and holistic understanding of the material. The goal was to capture first impressions, intuitive insights, and resonant elements related to the experiences of HCPs [23,24].

### Structural Analysis

**Figure 1:**
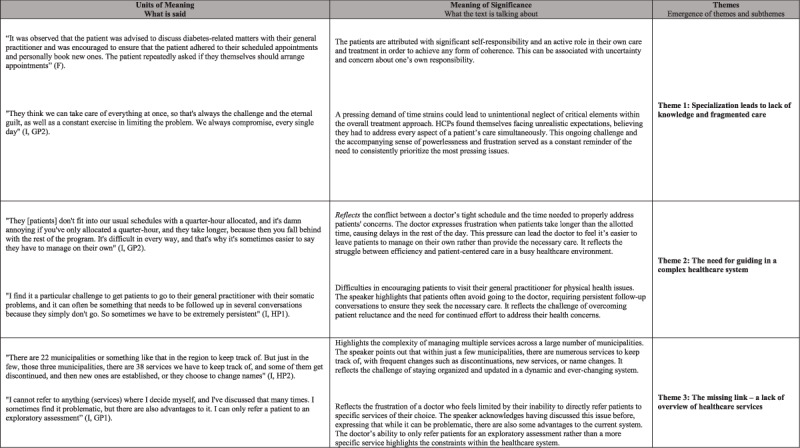
Examples of structural analysis process.

In this phase, the text was systematically broken down to identify units of meaning, leading to emerging patterns and preliminary themes. A dialectical process between explanation and understanding was employed to deepen interpretation and refine thematic categories [24,28] (Figure 1).

### Critical Interpretation and Discussion

The final phase involved interpreting the findings in light of relevant theory and existing literature. This enabled a more comprehensive and contextualized understanding of the data [28].

While validation from participants was not sought, given the aim of generating broader insights, the analysis and interpretations were continuously discussed within the supervisory team. This collaborative process supported analytical rigor and ensured that findings were relevant to clinical practice and aligned with scholarly standards [24,29].

## Description of the integrated care challenges

The analysis revealed three themes reflecting the experiences of managing treatment and care for individuals with schizophrenia and T2D. These themes included: *specialization leads to lack of knowledge and fragmented care, the need for guiding in a complex healthcare system*, and *the missing link – a lack of overview of healthcare services*. Subthemes connected to the main themes are illustrated in Table 1.

**Table 1:** Overview of themes and subthemes.


THEME	SUBTHEMES

**Theme 1: Specialization leads to lack of knowledge and fragmented care**	Collaboration Challenges Ineffective Collaboration Balancing Specialization and Generalization Education

**Theme 2: The need for guiding in a complex healthcare system**	People with Schizophrenia and T2D Frustration and Powerlessness Importance of Pushing Patients through the Complex Healthcare System Practical Support

**Theme 3: The missing link – a lack of overview of healthcare services**	Organizational Shortcomings Lack of Visibility and Overview Making Information and Services Visible Barriers in Participating and Tailored Support Need for Improved Visibility, Systematization, and Coordination


The first theme addressed HCPs experiences with challenges in cross-sector collaboration, which led to inadequate care and treatment for individuals with both schizophrenia and T2D. Specialization, incomplete information transfer, and a lack of knowledge about individuals’ “other” conditions, along with numerous appointments scattered across various healthcare sectors, significantly hindered the adoption of a holistic approach to care. This fragmentation meant that individuals were often left to coordinate their own care, navigating a system insufficiently prepared to support complex needs. Observations reinforced this, showing that patients frequently acted as the primary source of information, especially regarding their diabetes, as it was often not addressed unless the patient specifically highlighted the need. HCPs expressed their frustration and sadness at not being able to provide better and more integrated care. They felt powerless in many cases, caught between the demands of specialization and the unrealistic expectation that all HCPs should act as generalists in managing multifaceted health conditions. This tension was especially evident in the difficulties HCPs faced when balancing specialized expertise with the need for a more comprehensive, generalist approach.

While there was recognition that holistic care could be greatly enhanced through education, it was clearly that the system’s structural challenges posed significant barriers to such an approach. Economic and policy constraints within the healthcare system were particularly limiting, with policies that discharged patients from outpatient clinics prematurely, despite ongoing complex health issues. This left GPs with the burden of managing severe complications without adequate support. Additionally, time constraints, combined with the pressures of resource allocation, often meant that essential aspects of patient care were overlooked. As one GP noted, time pressures forced HCPs into constant compromises, never able to fully address every aspect of a patient’s care. This fragmentation, in turn, highlighted the unsustainable nature of expecting one HCP to meet the many needs of patients with schizophrenia and T2D.

The second theme described the frustration of HCPs when they encountered patients who did not fit into standard treatment frameworks. The rigid, time-limited nature of these frameworks further contributed to the inability of HCPs to provide proper follow-up or adopt a flexible, individualized approach to care. In these cases, patients’ complex needs were often insufficiently addressed, and HCPs expressed a clear need for more resources beyond the basic care model to facilitate better responsibility allocation. However, this need for more comprehensive support was complicated by the political and organizational decisions that defined the scope and division of responsibilities in healthcare. These decisions often left HCPs in positions where they lacked the authority or resources to effectively address the complex needs of their patients. This theme underscored the reality that patients with schizophrenia and T2D not only required more medical treatment but also needed additional practical support, something beyond the traditional scope of care provided by HCPs.

In the third theme, HCPs discussed the difficulties of navigating the fragmented landscape of healthcare services, which lacked cohesion due to organizational shortcomings. The lack of a clear overview of available services meant that patients often received care in an ad-hoc and random manner, further exacerbating the fragmented experience of care. This lack of systematization also meant that many individuals with schizophrenia and T2D were unaware of the services available to them, and HCPs often struggled with limited information about which services were best suited to their patients’ needs. A recurring call from HCPs was for better visibility and accessibility of services, with a specific push for diagnosis-specific services that could fit more effectively to this population. The current offerings within the healthcare system often did not meet the specific needs of individuals with co-occurring schizophrenia and T2D, prompting calls for tailored support and increased coordination across sectors.

The findings from these three themes underscore the deeply rooted challenges within the healthcare system when addressing the needs of individuals with complex comorbidities, such as schizophrenia and T2D. Furthermore, it shed light on the strain caused by a lack of integration between specialties, a fragmented healthcare system, and policy constraints, all of which limit the ability to provide comprehensive, coordinated care. These findings emphasize the urgent need for a more holistic approach to care, one that integrates cross-sector collaboration, improves communication, and allows for greater flexibility in treatment. In addition, the findings highlight the pressing need for political action and structural changes within the healthcare system to address these gaps in care and to ensure that patients with complex conditions receive the holistic support they require. Without such changes, the effectiveness of treatment for individuals with schizophrenia and T2D will remain severely constrained.

## Discussion

The findings of this study illustrated the complex and interrelated organizational challenges faced by HCPs when treating individuals with co-occurring schizophrenia and T2D. These challenges included a lack of integrated knowledge across psychiatric and somatic care domains, limited cross-sector collaboration, and systemic fragmentation. The results revealed how insufficient mutual understanding of professional roles and patient conditions often leads to suboptimal care delivery.

These observations resonate with existing literature. Studies have shown that HCPs frequently struggle to manage patients with multiple comorbidities, and this often results in prioritization dilemmas and uneven care quality [7]. Hultsjö et al. highlighted that diabetes care is frequently downgraded depending on the provider’s level of interest and expertise in somatic care. However, the same research emphasized that targeted education and increased knowledge can help mitigate this imbalance [17].

The organization of the Danish healthcare system plays a central role in shaping the challenges described by HCPs in this study. As outlined in the introduction, care for individuals with schizophrenia and T2D is divided across sectors: psychiatric services are delivered regionally, while somatic care, including diabetes treatment, is primarily managed in general practice, municipalities, or specialized hospital-based diabetes centers. This structural division between mental and somatic healthcare creates what participants described as fragmented care, with limited coordination, communication, and shared responsibility across professional boundaries. The lack of a formal integrated care pathway leaves patients to navigate between these services themselves, often without systematic support or care coordination. This aligns with existing literature on the Danish healthcare system, which highlights how specialization and sectoral divisions can inhibit holistic, person-centered care [8,30,31,32]. Moreover, the absence of shared digital records or designated care coordinators means that HCPs often work in parallel rather than collaboratively. These structural conditions both reflect and reinforce the siloed approach to healthcare, which this study’s findings suggest must be addressed to improve outcomes for individuals with complex, co-occurring conditions.

The findings further indicated that heightened specialization across sectors hinders cross-sectoral collaboration and leads to parallel rather than integrated care. Patients are often forced into the role of health advocates, compensating for the system’s lack of coordination. Bellass et al. similarly found that, despite recognizing the importance of collaboration, professionals in both mental and physical health services lacked the knowledge and structures needed to overcome siloed working practices [33]. Their study, like ours, emphasized that there is no one-size-fits-all solution, and that shared responsibility and mutual understanding of psychiatric and somatic needs are essential [33]. These findings point to a pressing need for systemic reforms aimed at strengthening cross-sectoral collaboration, formalizing care pathways, and ensuring that no patient is left to coordinate complex care journeys alone.

Moreover, patients rarely fit into standard treatment frameworks, which resulted in frustration and a sense of professional inadequacy among HCPs. This challenge was also connected to the broader structure of the healthcare system. Prioritization under time constraints was described as having a negative influence on decision-making, leading in some cases to undertreatment [7,34]. Limited resources and complex care needs contribute to unsystematic service delivery, often exacerbated by insufficient communication between professionals across different sectors. Better support structures, clear responsibility allocation, and system-wide coordination are needed to facilitate integrated care delivery.

A lack of visibility and overview of available healthcare services was another key challenge reported by participants. Gaps in systematization and reliance on chance encounters often determined whether patients accessed appropriate healthcare services. A recent professional presentation focusing on mental health improvements in Denmark confirmed these concerns, noting that services are not only lacking in availability but also in quality and interdisciplinary focus [35]. Blixen et al. also highlight the fragmented nature of existing services, which are often designed around individual treatment units and rarely integrated [8]. HCPs in our study advocated for more visible, accessible, and tailored services, particularly diagnosis-specific initiatives that can accommodate the needs of complex patient groups. In this context, the inclusion of peer workers was viewed as a potentially valuable strategy. Several studies have demonstrated that peer support can have positive effects on both mental and physical health outcomes, and may serve as a key element in developing more person-centered and integrated services [36,37,38,39]. Such roles could also help bridge the gap between sectors and contribute to more continuous and holistic care trajectories.

This study contributes to the understanding of HCPs’ experiences, applying a Ricoeur-inspired analytical framework to generate nuanced insights into their perspectives [24]. The findings are consistent with previous research, enhancing the study’s credibility. In line with Lincoln and Guba’s qualitative research criteria, attention was paid to credibility, transferability, dependability, and confirmability throughout the research process [40].

However, several methodological limitations should be acknowledged. The study’s small-scale nature and purposive sampling may limit generalizability. While every effort was made to reduce bias, factors related to participant selection, data collection, and analysis could have influenced the results. Some observations were conducted outdoors, which may raise concerns about transferability to clinical indoor settings and introduce a potential risk of cognitive bias. The COVID-19 pandemic also necessitated a shift in interview methods from in-person to online or telephone formats, which may have affected the depth or nuance of data collection. Lastly, the first author’s limited background in psychiatry could have influenced interpretation; however, this was mitigated through collaboration with experts and a thorough literature review.

Overall, these findings point toward a system in need of reform, where cross-sectoral alignment, increased visibility of services, and interdisciplinary understanding are prerequisites for truly integrated care.

## Lessons Learned

While the findings of this study offer valuable insights into improving care for individuals with schizophrenia and T2D, it is essential to consider how these practices can be scaled and applied more broadly. The following lessons highlight key strategies for enhancing integrated care and addressing the challenges identified:

1. **Standardized protocols and guidelines:**
  Implementing standardized care protocols across sectors can reduce fragmentation and support a more consistent, holistic approach. By sharing best practices and clinical guidelines, HCPs can coordinate more effectively, ensuring continuity of care and better patient outcomes.
2. **Cross-sector collaboration and policy reform:**
  Tackling the structural barriers to integrated care requires closer collaboration between healthcare providers and policymakers. This includes developing formal care pathways, fostering shared responsibility, and reallocating resources to support interdisciplinary training and joint practice models. Structural reforms are vital to ensure that patients are no longer left to navigate fragmented systems alone.
3. **Technology and telehealth integration:**
  Digital tools can enhance communication and coordination across sectors, particularly when embedded within a broader framework of shared records and cross-sector communication. Telehealth can also help alleviate time and resource constraints by improving accessibility, especially for patients with complex needs.
4. **Community engagement and patient empowerment:**
  Person-centered care depends on the involvement of patients, families, and communities in designing services. Empowering individuals through shared decision-making and incorporating peer support can improve engagement, treatment adherence, and overall care experiences. Partnerships with civil society and advocacy groups can further strengthen local support networks.

## Conclusions

This study offers important insights into the complexities of providing healthcare to individuals with co-occurring schizophrenia and T2D. Despite methodological limitations, including a small sample size and potential biases related to study design, the findings underscore the urgent need for integrated, patient-centered care.

Key recommendations include enhancing collaboration across sectors, improving communication and coordination among HCPs, and strengthening the visibility and systematization of services. Specialized training that bridges psychiatric and somatic domains is critical to equipping professionals with the necessary competencies.

Moreover, the inclusion of peer support roles and co-designed care pathways can improve service responsiveness and help patients better navigate complex care systems. Such efforts support a shift towards more holistic, tailored models of care.

Ultimately, this study calls for structural reforms that enable a more integrated and equitable healthcare system. Stakeholders, including healthcare leaders, policymakers, and community actors, must work together to implement these changes. By doing so, we can build a healthcare model that better supports individuals with complex and intersecting needs, and in turn, improve both clinical outcomes and quality of life.

## Appendix 1 – Interview guides from individual interviews and focus group

### Interview guide focus group

**Table:**

RESEARCH OBJECTIVES	OVERALL QUESTION	SUBSIDIARY QUESTIONS

1) How do patients with schizophrenia and diabetes, relatives, and healthcare professionals experience current practices regarding diabetes care?	What is your usual practice when a patient with schizophrenia also has diabetes? **For this first topic, I would like each of you to jot down a few keywords on a post-it note about the topic and then take turns sharing what you have written**. What are your immediate thoughts on the current practice for managing a diabetes diagnosis in psychiatry? **Again, feel free to individually jot down a few keywords on a post-it note, after which we’ll discuss them collectively**. Do you have a specific program or offer for these patients in psychiatry? Please elaborate (what do they consist of?) **Group discussion**. How could you envision or wish for a course of treatment to best support patients who have both schizophrenia and diabetes? **For this topic, I’d like each of you to once again jot down a few keywords on a post-it note and then take turns sharing what you have written**.	Do you do anything special for this patient group that is different? How do you experience that the patient is supported in having both a mental and a physical illness? Why/why not? Could you/I elaborate on what you/they are saying? Could you/I possibly add a few more words? In what way does it manifest? What makes it difficult/not difficult? Can you/I tell more about why you think that? Do you find that there is collaboration across sectors such as with the hospital or general practitioner? Please elaborate? How does it work? Why/why not? Is there something missing in your opinion? Hvordan kunne det effektueres? How could it be implemented? How could it benefit the patient? Who should have ”responsibility” in the patient process? Could it also benefit you as healthcare professionals? How/why? Please elaborate?

2) What challenges and needs do patients with schizophrenia and diabetes, relatives, and healthcare professionals express in achieving improved diabetes care?	What do you find most challenging for the patient with schizophrenia in having to manage diabetes at the same time? **Feel free to individually jot down a few keywords on a post-it note, after which we’ll discuss them collectively**. What is your experience when patients with schizophrenia also have diabetes – do you do anything differently? **Group discussion**. What do you find occupies the patient with schizophrenia and diabetes the most? And is diabetes even addressed by you/them or the patient in the conversation? **Further discussion in the group**. How could there be more focus on both diseases in psychiatry in the future? **For this, please jot down a few keywords on a post-it note individually, and then take turns sharing what you have written**. What other needs could you/I imagine or wish for in relation to patients with schizophrenia achieving improved diabetes care? **Feel free to individually jot down a few keywords on a post-it note (think outside the box), after which we’ll discuss them collectively**.	Why can it be a challenge? Could you/I possibly add a few more words? What is particularly challenging for this patient group compared to others? Why/why not? Could you/I tell a little more about what is the focus of your conversations? Can you/I elaborate? Why do you/they think that? What could be the reason in your/their opinion? Why do you/they address it/not address it in the conversation? Can you/I add a few more words? What factors influence it (time, lack of knowledge, other)? What is needed or should something be done? Could you/I elaborate more on how? Is it a shared responsibility (role distribution)? Where/what do you think are your and the patients’ responsibilities? Is it something you could see as an advantage for the patients? Why/why not? What would it require from you/them? Why? How? Could you/I elaborate more? Do you/they have experience with something that has worked well/less well? Would you/I like to add a few more words to that?

	As a conclusion, I’d like to ask if there’s anything else you didn’t get to share or would like to supplement with? **Plenum**.	If necessary, I may need to come back to you to get more information about your experiences?

### Interview guide individual semi-structured interviews with HCPs

**Table:**

RESEARCH OBJECTIVES	OVERALL QUESTION	SUBSIDIARY QUESTIONS

	**Introduction: Brief information on how the interview will proceed – informed consent, and recording** Would you start by briefly introducing yourself? Your age, seniority, how long you have been working in this area?	

1) How do patients with schizophrenia and diabetes, relatives, and healthcare professionals experience current practices regarding diabetes care?	**As you have been informed in the materials sent, our project focuses on citizens who have both schizophrenia and type 2 diabetes**. Do you have experience with this group of patients? Could you try to tell me about your experiences? Do you have a specific program or offer for these citizens? Please elaborate (what do they consist of?) Who is involved (staff) in these programs/offers or citizens? Please elaborate. Can you tell me a bit about how a typical program for citizens with schizophrenia and type 2 diabetes proceeds here?	What you’re saying… Why? How? Can you elaborate? Can you put more words on it? In what way? How does it manifest? Can you tell me more about why you think that way? To make sure I understand what you’re saying… Is it correctly understood that… Can you elaborate on how/why? Is there cooperation across sectors, e.g., with the hospital or general practitioner? Can you elaborate more on what the basis is for the current treatment? Can you tell me more about what is often or particularly focused on in the meeting/conversation?

2) What challenges and needs do patients with schizophrenia and diabetes, relatives, and healthcare professionals express in achieving improved diabetes care?	**Schizophrenia and diabetes are two chronic diseases that can affect each other**. How do you perceive that both conditions are addressed when citizens with schizophrenia and type 2 diabetes are seen by you? Do you find support and treatment for this group of citizens particularly challenging? Is it your experience that you have a special approach to care and treatment when you see citizens with schizophrenia and diabetes? If we assume that anything could be possible, is there anything you would like to do? Or do differently compared to usual practice?	Why/why not? Can you tell me more about it? Do you have a specific situation/experience that has made an impression on you? If yes, would you tell me more about this and what significance it had for you? Why do you think it’s like that? Can you tell me a bit more about what you’re articulating? What, in your experience, is the reason for this? Please elaborate.

	As a conclusion, I would like to hear if you have anything else you didn’t get to mention or would like to add? If necessary, may I contact you again to learn more about your experiences? Lastly, I would like to hear about your experience of being interviewed by me, and if there was anything I could do differently next time? **If you think of anything after we have finished the interview, you are more than welcome to contact me**.	
